# A Subset of Odorant Receptors from the Desert Locust *Schistocerca gregaria* Is Co-Expressed with the Sensory Neuron Membrane Protein 1

**DOI:** 10.3390/insects10100350

**Published:** 2019-10-17

**Authors:** Pablo Pregitzer, Xingcong Jiang, René-Sebastian Lemke, Jürgen Krieger, Jörg Fleischer, Heinz Breer

**Affiliations:** 1 Institute of Physiology (230), University of Hohenheim, 70599 Stuttgart, Germany; jiangxingcong@126.com (X.J.); breer@uni-hohenheim.de (H.B.); 2 Department of Evolutionary Neuroethology, Max Planck Institute for Chemical Ecology, 07745 Jena, Germany; 3 Department of Animal Physiology, Institute of Biology/Zoology, Martin Luther University Halle-Wittenberg, 06120 Halle (Saale), Germany; rene-sebastian.lemke@zoologie.uni-halle.de (R.-S.L.); juergen.krieger@zoologie.uni-halle.de (J.K.); joerg.fleischer@zoologie.uni-halle.de (J.F.)

**Keywords:** insect, locust, olfaction, odorant receptor, SNMP1, in situ hybridization

## Abstract

In the desert locust *Schistocerca gregaria* (*S. gregaria*), pheromones are considered to be crucial for governing important behaviors and processes, including phase transition, reproduction, aggregation and swarm formation. The receptors mediating pheromone detection in olfactory sensory neurons (OSNs) on the antenna of *S. gregaria* are unknown. Since pheromone receptors in other insects belong to the odorant receptor (OR) family and are typically co-expressed with the “sensory neuron membrane protein 1” (SNMP1), in our search for putative pheromone receptors of *S. gregaria*, we have screened the OR repertoire for receptor types that are expressed in SNMP1-positive OSNs. Based on phylogenetic analyses, we categorized the 119 ORs of *S. gregaria* into three groups (I–III) and analyzed a substantial number of ORs for co-expression with SNMP1 by two-color fluorescence in situ hybridization. We have identified 33 ORs that were co-expressed with SNMP1. In fact, the majority of ORs from group I and II were found to be expressed in SNMP1-positive OSNs, but only very few receptors from group III, which comprises approximately 60% of all ORs from *S. gregaria*, were co-expressed with SNMP1. These findings indicate that numerous ORs from group I and II could be important for pheromone communication. Collectively, we have identified a broad range of candidate pheromone receptors in *S. gregaria* that are not randomly distributed throughout the OR family but rather segregate into phylogenetically distinct receptor clades.

## 1. Introduction

Chemical signals from the environment trigger important behaviors in insects, such as foraging, reproduction, or aggregation [1,2]. The recognition and primary responses to suitable compounds take place in cuticular hair-like structures, called sensilla, on the surface of chemosensory organs. Within such sensilla, olfactory sensory neurons (OSNs) project their dendritic process into the fluid-filled sensillar cavity, and the dendritic membrane is endowed with receptors that render these neurons responsive to distinct substances [1,3]. Pheromones, signal molecules for intraspecific communication, are detected by specialized OSNs that are usually characterized not only by the expression of pheromone receptors and the olfactory receptor co-receptor (Orco), but also by the “sensory neuron membrane protein 1” (SNMP1) [4,5,6,7,8,9]. SNMP1 was initially discovered in the noctuid moth *Antheraea polyphemus* as a major protein component in membranes of pheromone-responsive OSNs [10,11,12]. More recent studies have shown that SNMP1 is located in close proximity to odorant receptor (OR) proteins and seems to be important for the high sensitivity of pheromone-responsive neurons [13,14]. Moreover, studies on the vinegar fly *Drosophila melanogaster* (*D. melanogaster*) have demonstrated that SNMP1 plays an essential role in the detection of the sex pheromone cis-vaccenyl acetate [4,15,16].

In contrast to moth and fly species, for the hemimetabolous locusts, including the desert locust *Schistocerca gregaria* (*S. gregaria*), our knowledge about the identity of pheromone compounds and the molecular elements involved in pheromone recognition is very sparse. However, a more detailed insight into the molecular basis of pheromone signaling in the desert locust is of great interest since this species is well known for its ability to form giant swarms that threaten agricultural crops in Northern Africa and the Middle East [17,18]. Importantly, several processes underlying swarm formation, such as transition from the solitarious to the gregarious phase, massive reproduction and aggregation [2,19], are based on intraspecific communication partly mediated by pheromones detected by specific neurons in antennal sensilla [20,21,22,23].

As a first step in elucidating the molecular mechanisms underlying the intraspecific chemical communication in the desert locust and to identify putative pheromone receptors, we have recently determined the repertoire of odorant receptors (ORs) in *S. gregaria* [24], since in other insects, members from the OR family of olfactory receptors mediate the detection of pheromones [25]. The analyses have led to the identification of 119 OR types expressed in antennal tissue of *S. gregaria* [24].

Based on the findings from dipterans and lepidopterans demonstrating that SNMP1 is expressed in pheromone-responsive neurons and is important for sensitive pheromone detection [4,14,15,16,26], OR types co-expressed with SNMP1 are considered as candidate pheromone receptors. In a previous study, we have found that most members of a small group of ORs in *S. gregaria* (the so-called “b-ORs”) and also a few other OR types are in fact co-expressed with SNMP1 [24]. In the course of our ongoing efforts to identify potential pheromone receptors of the desert locust, we set out for a more comprehensive search for OR subtypes of *S. gregaria* (SgreORs) that are co-expressed with SNMP1 in antennal OSNs.

## 2. Materials and Methods

### 2.1. Phylogenetic Analyses

Analyses of the phylogenetic relatedness of locust OR sequences were based upon a neighbor-joining phylogenetic tree described in more detail in [24]. For generating this tree, the deduced amino acid sequences of 117 SgreORs out of the 119 previously identified SgreOR sequences were utilized (two SgreORs that represented only very short and partial OR sequences were excluded). Besides these 117 SgreORs, this tree also comprises 138 OR sequences from the migratory locust *Locusta migratoria* (*L. migratoria*); the latter are designated as LmigOrs. The tree was rooted using sequences from several insect species (*S. gregaria*, *L. migratoria*, *Manduca sexta*, *D. melanogaster* and *Anopheles gambiae*) coding for the odorant receptor co-receptor Orco. According to their relatedness, the locust OR types were divided into three phylogenetic lineages (group I–III). Group I (previously named “basal OR” or “b-OR” group due to its close proximity to the Orco sequences located at a basal position in the tree) includes 9 SgreOR types. A monophyletic group comprising 38 SgreORs that emanate from a node of the phylogenetic tree supported by a bootstrap value of 85 was classified as group II. The remaining 70 SgreORs that emanate from another node with a bootstrap value of 59 were assigned to group III. 

### 2.2. Animals and Preparation of Antennae

Gregarious desert locusts (*Schistocerca gregaria*) were purchased from Bugs-International GmbH (Irsingen/Unterfeld, Germany) and were temporarily kept until usage. Alternatively, gregarious desert locusts were reared as described previously [27].

Antennae of adult males and adult females were dissected using autoclaved surgical scissors and were immediately frozen in liquid nitrogen. Antennae were used directly or stored at −70 °C before subsequent RNA extraction. 

### 2.3. Reverse Transcription PCR Amplification for Generating Riboprobes

Total RNA was extracted from frozen antennae using Trizol reagent (Thermo Fisher Scientific, Waltham, MA, USA) following the protocol recommended by the manufacturer. 100 μg of the extracted total RNA was subsequently purified using the Dynabeads mRNA purification kit (Thermo Fisher Scientific) according to the instructions of the supplier to obtain poly (A)^+^ RNA. The isolated poly (A)^+^ RNA was reversely transcribed to cDNA in a total volume of 20 μL with SuperScriptTM III Reverse Transcriptase (Thermo Fisher Scientific) as recommended by the manufacturer. PCR conditions used in subsequent PCR experiments were set as follows: 94 °C for 100 s, then 20 cycles with 94 °C for 30 s, 50–60 °C for 30 s (thereby, the annealing temperature was reduced by 0.5 °C per cycle) and 72 °C for 2 min. Using the conditions of the last cycling step (annealing temperature was 40–50 °C), further 20 cycles were run, followed by a 15-min incubation at 72 °C. The oligonucleotide primers used for these PCR experiments are documented in Table S1. Amplified PCR products were cloned into pGEM-T vector (Thermo Fisher Scientific) and sequenced prior to the subsequent in vitro transcription.

### 2.4. Synthesis of Riboprobes for In Situ Hybridization

Linearized pGEM-T plasmids containing SgreOR-encoding sequence fragments or sequences coding for SNMP1 [24,28] were utilized to synthesize antisense riboprobes labeled with digoxigenin or biotin, using the T7/SP6 RNA transcription system (Roche Diagnostics, Mannheim, Germany). The synthesis procedure conformed to the protocol provided by the manufacturer.

### 2.5. Fluorescence In Situ Hybridization (FISH)

Dissected antennae of adult *S. gregaria* were embedded in Tissue-Tek O.C.T. Compound (Sakura Finetek Europe, Netherlands). Longitudinal cryosections (12 µm thick) through antennae were prepared at −21 °C with a Jung CM300 cryostat (Leica Microsystems, Bensheim, Germany) and were thaw-mounted on SuperFrost Plus slides (Thermo Fisher Scientific). 

FISH experiments were carried out as described previously [24,29,30]. Briefly, the cryosections were fixed in 4% paraformaldehyde (in 0.1 M NaHCO_3_, pH 9.5) at 4 °C for 22 min, followed by a series of treatments at room temperature comprising a wash for 1 min in phosphate-buffered saline (PBS, 0.85% NaCl, 1.4 mM KH_2_PO_4_, 8 mM Na_2_HPO_4_, pH 7.1), an incubation for 10 min in 0.2 M HCl, another wash for 1 min in PBS, an incubation for 10 min in acetylation solution (0.25% acetic anhydride freshly added in 0.1 M triethanolamine) and three washes in PBS (3 min each). Sections were incubated in pre-hybridization solution [5× SSC (0.75 M NaCl, 0.075 M sodium citrate, pH 7.0) and 50% formamide] for 15 min at 4 °C. Subsequently, the sections were hybridized in 100 μL hybridization solution 1 (50% formamide, 2× SSC, 10% dextran sulphate, 0.2 mg/mL yeast t-RNA, 0.2 mg/mL herring sperm DNA) supplemented with the relevant digoxigenin- and biotin-labeled riboprobes. Alternatively, sections were pre-hybridized for 1 h at 60 °C in hybridization buffer 2 (50% formamide, 5× SSC, 50 μg/mL heparin and 0.1% Tween-20). A volume of 100 μL hybridization buffer 2 supplemented with the relevant RNA probes was evenly applied onto each slide with adhered tissue sections. Next, a coverslip was placed on top and slides were incubated at 60 °C overnight (18–20 h) in a box equipped with paper towels soaked with 50% formamide. On the following day, slides were washed twice for 30 min in 0.1× SSC at 60 °C; then each slide was treated with 1 mL 1% blocking reagent (Roche Diagnostics) in TBS (100 mM TRIS, pH 7.5, 150 mM NaCl) for 40 min at room temperature in a box to keep the sections moist. Visualization of hybridized digoxigenin-labeled riboprobes was performed by using an alkaline phosphatase-conjugated antibody against digoxigenin in combination with HNPP/Fast Red (Roche Diagnostics). To visualize probes labeled with biotin, streptavidin conjugated to horseradish peroxidase was utilized together with tyramides coupled to fluorescein (TSA kit, Perkin Elmer, Waltham, MA, USA). The slides were analyzed with a Zeiss LSM510 Meta laser scanning microscope (Carl Zeiss Microscopy, Jena, Germany) and the acquired confocal image stacks were further processed by ZEN 2009 software (Carl Zeiss Microscopy). The images presented in Figures 2–5 and in Figure S1 depict projections of several optical planes chosen from continuous confocal image stacks. To enhance data clarity, the images were only adjusted in brightness and contrast. Antennal sections of both males and females were treated under identical experimental conditions with the riboprobes generated for SNMP1 and the relevant SgreORs; no obvious sex-dependent differences regarding the staining intensity as well as the labeling pattern were found. Therefore, only images acquired from antennal sections of males are presented in Figures 2–5 and in Figure S1.

### 2.6. RNA Extraction and Reverse Transcription PCR for Comparing SgreOR Expression in Males Versus Females

To investigate a potentially sex-dependent expression of the group II SgreOR types co-expressed with SNMP1, the entire antennae from 10 male and 10 female, sexually mature individuals were removed surgically. After freezing the antennae immediately in liquid nitrogen, RNA was extracted utilizing Trizol reagent as recommended by the supplier. Next, poly(A)^+^ RNA was extracted with the Dynabeads mRNA purification kit following the manufacturer’s protocol. Finally, poly(A)^+^ RNA samples were eluted with 22 µl H_2_O and RNA concentrations were measured by means of an Epoch microplate spectrophotometer (BioTek, Winooski, VT, USA). Synthesis of first strand cDNA was carried out with 250 ng of poly(A)^+^ RNA, 2 µL 50 µM oligo(dT)20 primer (Thermo Fisher Scientific), 2 µL 10 mM 2’-deoxynucleoside 5’-triphosphate (dNTP) solution mix (New England Biolabs, Ipswich, MA, USA) and RNase-free H_2_O. Following a 5-min incubation at 65 °C, 8 µL 5× SSIV Buffer (Thermo Fisher Scientific), 2 μL 100 mM 1,4-dithiothreitol (DTT; Thermo Fisher Scientific), 2 µL RNaseOut (Thermo Fisher Scientific) and 1 µL Superscript IV Reverse Transcriptase (Thermo Fisher Scientific) were added. Synthesis of cDNA was conducted at 52 °C for 50 min followed by 10 min at 80 °C. In parallel, control samples were prepared for which no reverse transcriptase was added. 

For the subsequent amplification of sequences encoding actin (GenBank accession number: AEV89776) or the OR types SgreOR84-86, SgreOR88, SgreOR90-97, SgreOR101-103, SgreOR109-114, SgreOR116 and SgreOR118 in PCR experiments, the oligonucleotide primers given in Table S2 were utilized. PCR reactions were prepared with 41 µL H_2_O, 5 µL 10× Titanium Taq PCR Buffer (Takara Bio, Saint-Germain-en-Laye, France), 0.5 µL of each primer (100 µM), 1 µL 10 mM dNTP solution mix, 2 µL first strand cDNA and 0.5 µL 50× Titanium Taq DNA Polymerase (Takara Bio). Thermocycling was conducted using the following parameters: 1 min at 97 °C succeeded by 35 cycles with 97 °C for 30 s and 3 min at 68 °C. After the last cycle, samples were incubated at 68 °C for 3 min. The resulting PCR products were visualized using agarose gels supplemented with ethidium bromide. PCR amplicons of the predicted molecular size were extracted using the Monarch DNA Gel Extraction Kit (New England Biolabs) and sent to GATC Biotech (Konstanz, Germany) for sequencing.

## 3. Results

### 3.1. Categorization of SgreORs Based on Phylogenetic Analyses

**Figure 1:**
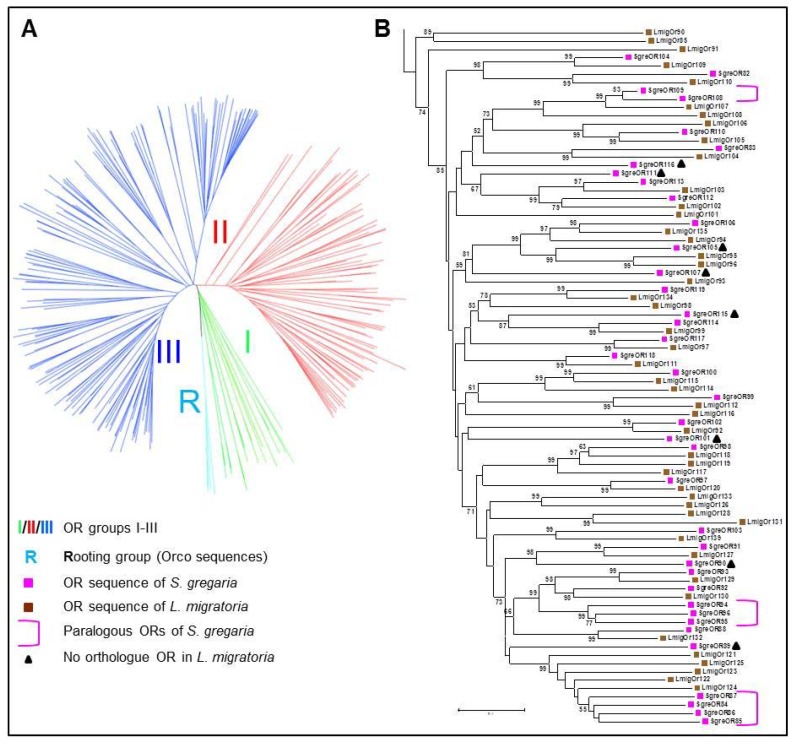
Phylogeny of *S. gregaria* (SgreORs). (**A**) Phylogenetic tree comprising ORs from *S. gregaria* (SgreOR) and *L. migratoria* (LmigOr). The tree was modified from [32] and is based on the original phylogenetic tree described in [24]. For simplification, a schematic representation of the phylogenetic tree is shown. The SgreORs were classified in three groups (I–III) indicated by the color code (I = green, II = red, III = blue). The outgroup used for rooting (Orco sequences, in light blue) is designated by “R”. (**B**) Rectangular representation of the branch of the phylogenetic tree harboring group II SgreORs and the corresponding LmigOrs. The scale bar indicates 10% difference. The numbers in the tree specify bootstrap support values based on 1000 replicates (only values above 50% are given). SgreORs and LmigOrs are indicated by pink or brown squares, respectively. Pink brackets denote paralogous SgreOR clusters or pairs. Black triangles indicate SgreOR types without an orthologous sequence in *L. migratoria*.

In this study, we extended our recent efforts to identify candidate pheromone receptors of *S. gregaria* [24] and examined a larger number of OR types for a possible co-expression with SNMP1, a marker for pheromone-sensitive OSNs. As a first step, SgreORs were categorized into groups according to our previous phylogenetic analyses [24] utilizing the deduced amino acid sequences of 117 SgreOR types [24], 138 ORs from the related locust species *Locusta migratoria* (LmigOrs) [31] and several sequences for Orco from distinct insect species. Based on these analyses, the SgreORs segregated into three groups (group I–III) that are highlighted in green (group I), red (group II) or blue (group III) color in the phylogenetic tree depicted in Figure 1A. In a recent study, we have primarily analyzed SgreORs of group I (previously called “b-OR” group) and found co-expression of most group I ORs with SNMP1; yet, testing several SgreORs that do not belong to group I, five receptors were also found to be co-expressed with SNMP1 [24]. According to the above described phylogenetic analyses, these five SgreORs are members of group II; this finding led us to investigate the SgreORs of group II in more detail.

The arrangement of SgreORs and the corresponding ORs from *L. migratoria* [31] in the phylogenetic tree (depicted in a rectangular representation in Figure 1B) revealed several interesting features in group II that are rare or absent in group I. Notably, while for eight of the nine members of group I, an orthologue exists in *L. migratoria* [24], out of the 38 group II SgreORs, 17 members apparently lack an orthologue in *L. migratoria* (indicated by the pink brackets and the black triangles in Figure 1B). Interestingly, nine of them have paralogues (Figure 1B), a phenomenon that does not exist in group I SgreORs [24]. Thus, according to our phylogenetic analyses, the SgreORs from group II can be further subdivided into three categories: the first category comprises nine SgreOR types that form three small clusters or pairs of paralogues; the second category includes eight non-paralogous SgreORs that lack an orthologue in *L. migratoria* and the third category consists of 21 SgreOR types with an orthologue in *L. migratoria* (Figure 1B). Therefore, in our search for SgreORs from group II that are co-expressed with SNMP1, we have investigated a potential co-expression with SNMP1 for members of these distinct categories separately by conducting two-color fluorescence in situ hybridization (FISH) experiments with a biotin-labeled riboprobe for SNMP1 and digoxigenin-labeled riboprobes for selected SgreORs of group II.

### 3.2. Expression of SgreORs and SNMP1

**Figure 2:**
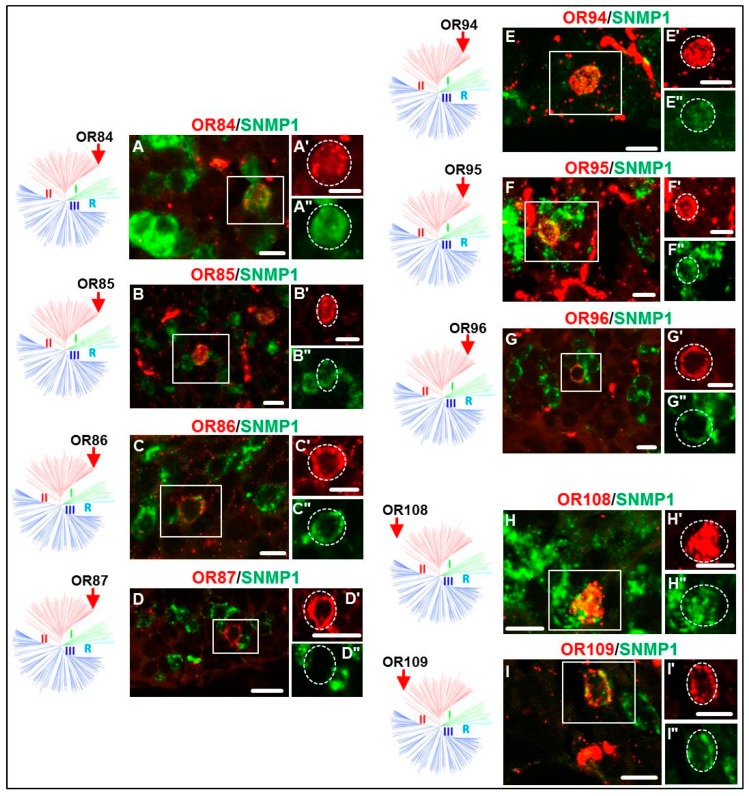
Expression of SNMP1 and group II SgreORs with paralogous sequences. (**A–I**) Expression of SNMP1 and SgreOR types of the three clusters/pairs comprising paralogues (SgreOR84-87, SgreOR94-96 and SgreOR108-109) was visualized by two-color FISH on antennal sections using specific antisense riboprobes for SgreORs (red fluorescence) and SNMP1 (green fluorescence). (**A’–I’, A’’–I’**’) Higher magnifications of single fluorescence channel images depicting the regions indicated by the white squares in A to I. The broken lines highlight the position of cells that express the defined OR types. With the exception of SgreOR87, all analyzed SgreORs are co-expressed with SNMP1. Scale bars, 10 μm. On the left side of the images from FISH experiments, red arrows in the schematic representations of the phylogenetic tree indicate the position of the relevant SgreORs.

**Figure 3:**
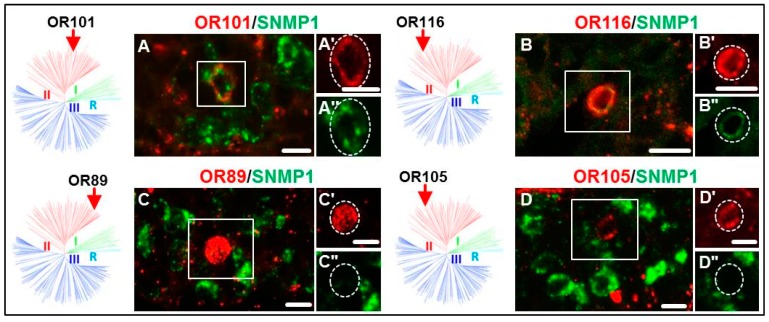
Expression of SNMP1 and group II SgreORs without an orthologous sequence. (**A–D**) Two-color FISH experiments on antennal sections from *S. gregaria* with specific antisense riboprobes for SNMP1 (green) and selected SgreORs of group II that lack an orthologue in *L. migratoria* (SgreOR101, SgreOR116, SgreOR89 and SgreOR105, red). (**A’–D’, A’’–D’’**). High magnification images of the boxed areas in **A–D** are shown in either the red or the green fluorescence channel. Cells expressing the relevant SgreOR types are circumscribed by the dashed lines. In contrast to SgreOR89 and SgreOR105, SgreOR101 and SgreOR116 are co-expressed with SNMP1. Scale bars, 10 μm. The position of the respective SgreOR types is denoted by arrows in the schematic representations of the phylogenetic tree depicted to the left of the images from FISH experiments.

**Figure 4:**
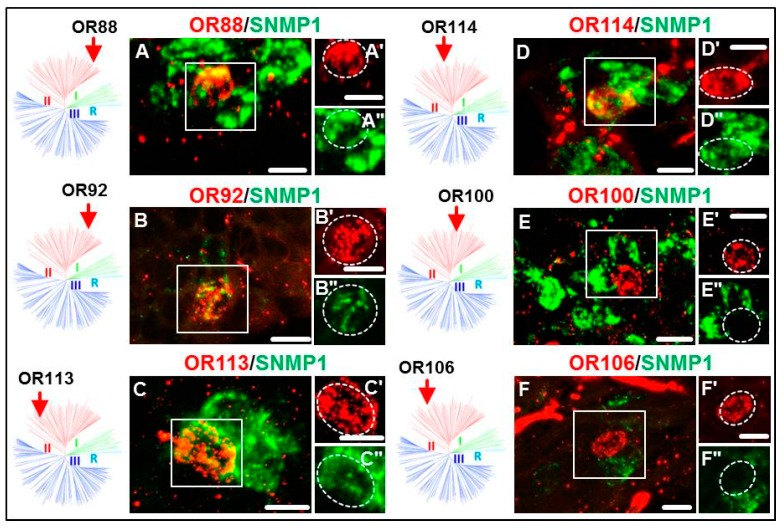
Expression of SNMP1 and group II SgreORs with an orthologues sequence. (**A–F**) Two-color FISH on sections through the antenna of *S. gregaria* incubated with antisense probes for SNMP1 (green) and selected SgreOR types (SgreOR88, SgreOR92, SgreOR113, SgreOR114, SgreOR100 and SgreOR106) that have an orthologue in *L. migratoria*. (**A’–F’, A’’–F’’**). Higher magnifications of the boxed regions in **A–F** are depicted for the red and the green fluorescence channel. The broken lines denote cells that express the relevant SgreOR types. While cells positive for SgreOR88, SgreOR92, SgreOR113 or SgreOR114 co-express SNMP1, cells expressing SgreOR100 or SgreOR106 are negative for SNMP1. Scale bars, 10 μm. The arrows in the schematic representations of the phylogenetic tree mark the position of the relevant SgreORs.

At first, ORs of the paralogous clusters/pairs (SgreOR84-87, SgreOR94-96 and SgreOR108-109) were analyzed. In our previous study [24], it was found that three of these receptors (SgreOR84, SgreOR86 and SgreOR94) are co-expressed with SNMP1 in antennal OSNs. The results of FISH experiments on longitudinal sections through antennae demonstrated that eight out of the nine members (~89%) of this category were co-expressed with SNMP1; only SgreOR87 was not co-expressed with SNMP1 (Figure 2). Next, we analyzed non-paralogous SgreORs from group II that lack an orthologous sequence in *L. migratoria*. Scrutinizing six out of the eight ORs from this category, we observed co-expression with SNMP1 for SgreOR90, SgreOR101, SgreOR111 and SgreOR116, whereas two of these receptors (SgreOR89 and SgreOR105) were found to be not co-expressed with SNMP1. The images of the corresponding FISH experiments for some of these ORs are exemplarily depicted in Figure 3. Finally, we analyzed SgreORs from group II with an orthologous sequence in *L. migratoria*. From 21 SgreORs of this category, 18 were analyzed by two-color FISH. The results of these approaches are shown for some of these ORs in Figure 4. These experiments revealed that 12 of these SgreORs were co-expressed with SNMP1, whereas six of them were not co-expressed. Thus, regarding the two categories without paralogous receptors, the percentage of the analyzed SgrORs co-expressed with SNMP1 is in both cases about 67% (four out of six and 12 out of 18, respectively). In summary, analyzing 33 out of the 38 SgreORs from group II, 24 of them (>72%) were found to be co-expressed with SNMP1, indicating that most group II SgreOR types are expressed in SNMP1-positive antennal OSNs. Group II SgreORs that are co-expressed with SNMP1 and have not been depicted in Figure 2, Figure 3 and Figure 4 are shown in Figure S1; co-expression of OR102 and OR111 with SNMP1 was demonstrated previously [32].

**Figure 5:**
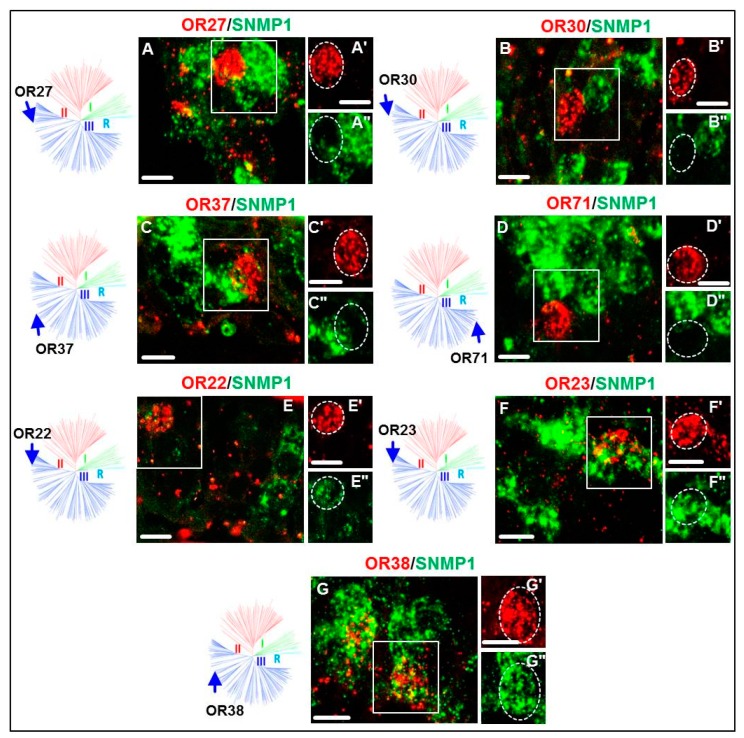
Expression of SNMP1 and group III SgreORs. (**A–G**) Expression of distinct group III OR types as well as SNMP1 was visualized by two-color FISH on antennal sections from *S. gregaria* using specific antisense riboprobes for SNMP1 (green) and SgreORs (red). (**A’–G’, A’’–G’’**) High magnification images of the boxed areas in **A–G** are shown in either the red or the green fluorescence channel. The dashed lines highlight cells that express the defined SgreOR types. Scale bars, 10 μm. The position of the respective SgreOR types is denoted by arrows in the schematic representations of the phylogenetic tree.

Within the OR repertoire of *S. gregaria,* group III comprises by far the largest number (70) of receptors. In order to explore a potential co-expression with SNMP1 in antennal OSNs, we have previously [24] analyzed 18 SgreORs that in the present study turned out to belong to group III. None of them was co-expressed with SNMP1 [24]. These findings might suggest that SgreORs from group III are generally not co-expressed with SNMP1. To scrutinize this notion in more detail, we examined additional 24 SgreORs from group III for a potential co-expression with SNMP1 by two-color FISH experiments on antennal sections. The resulting images for a number of these ORs are depicted in Figure 5. Among these receptors, only three of them (SgreOR22, SgreOR23 and SgreOR38) were observed to be co-expressed in OSNs with SNMP1. Thus, combining the results of our present and previous [24] studies, the vast majority (39) of the 42 analyzed group III SgreORs were not co-expressed with SNMP1. 

A compilation of the results acquired by the FISH experiments, including the findings of our previous study [24], is presented in Table 1. From the 119 SgreOR sequences, we have analyzed 83. Collectively, 33 of these SgreORs (~40%) are in fact co-expressed with SNMP1, including receptor types from all three groups: 6 from group I, 24 from group II and 3 from group III. Hence approximately 73% of the identified SgreORs types co-expressed with SNMP1 fall into group II. Based on their co-expression with SNMP1, a marker for pheromone-responsive OSNs, they may be considered as candidate pheromone receptors.

### 3.3. Expression of Group II ORs Co-Expressed with SNMP1 in Males and Females

**Figure 6:**
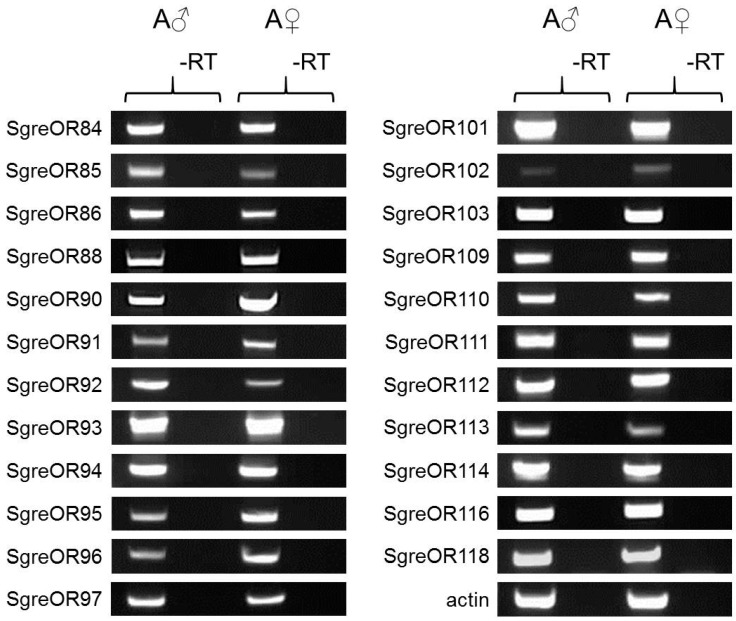
Analyses of a potentially sex-dependent expression of SgreORs of group II that are co-expressed with SNMP1. For 23 SgreOR types from group II that are co-expressed with SNMP1, expression in the antenna of male (A♂) and female (A♀) adult desert locusts was assessed by PCR experiments using receptor-specific primer pairs (listed in Table S2) and antennal cDNA. The resulting PCR products depicted have a predicted molecular size. Their identity was scrutinized by sequencing analyses, demonstrating that they encoded for the relevant SgreORs. In experiments with a negative control template (-RT), for which no reverse transcriptase was added to the cDNA synthesis reaction with antennal RNA, no amplicons of the expected molecular weight were observable.

For lepidopteran species, it has previously been shown that receptors for certain pheromones, especially for sex pheromones, are often expressed in a sex-specific or at least sex-biased manner [7,9]. To approach the question whether SgreOR types co-expressed with SNMP1 are expressed in a sex-biased manner, similar to several lepidopteran pheromone receptors, we have performed semi-quantitative reverse transcription PCR studies with cDNAs derived from antennae of either male or female adult *S. gregaria*. The results for several SgreORs from group II are depicted in Figure 6. Although slight differences can be seen, the overall picture indicates for all analyzed SgreOR types a similar expression level in males and females; an observation reminiscent of recent results found for SgreORs from group I [33].

## 4. Discussion

In this study, attempts were made to identify candidate pheromone receptors from the desert locust *S. gregaria* by screening the large repertoire of SgreORs for co-expression with SNMP1 that is considered a marker for pheromone-responsive OSNs [4,5]. Together with a recent study [24], 83 SgreORs (representing ~70% of the known SgreOR repertoire) were analyzed and among them, 33 OR types were found to be co-expressed with SNMP1 (Table 1). Currently, it cannot be excluded that the total number of SgreORs co-expressed with SNMP1 is somewhat higher since 36 SgreORs could not be analyzed yet. However, since almost all of these ORs belong to group III, which comprises SgreORs that are usually not co-expressed with SNMP1 (Table 1), the absolute number of SgreOR types co-expressed with SNMP1 may not be substantially higher than 33. 

So far, the repertoire of ORs that are co-expressed with SNMP1 has been sparsely analyzed in other insects. In the dipteran species *D. melanogaster*, out of the 37 OR types that are expressed in the antenna [34], only nine are co-expressed with SNMP1 [4]. Consequently, in spite of a clearly smaller antennal OR repertoire in *D. melanogaster*, the desert locust has a comparatively large number of ORs that are co-expressed with SNMP1. This finding raises the question why the number of ORs co-expressed with SNMP1 is that high in *S. gregaria*. In this regard, it is important to note that locusts are characterized by a phase polyphenism and are well known for their ability to form giant swarms [18,35]. For aggregation and subsequent swarm formation in locusts, intraspecific chemical communication via pheromone substances is considered to be of crucial significance [2,35]. Moreover, a number of chemicals have been proposed to be involved in governing gregarization/phase transition, reproduction, coordinated oviposition and maturation synchronization in locusts [2,35,36,37]. Thus, the relatively high number of SNMP1-co-expressed candidate pheromone receptors in *S. gregaria* may further substantiate the concept that in locusts, a large variety of behaviors and physiological processes rely on intraspecific communication via a broad spectrum of chemical signals. However, some of the SgreOR types co-expressed with SNMP1 could be involved in the reception of non-pheromonal compounds. In particular, since a few ORs from *D. melanogaster,* namely Or19a or Or83c, that are co-expressed with SNMP1 [4,34] are activated by odorants related to food sources [38,39]. Therefore, functional analyses are required to unravel the ligand spectrum of receptors co-expressed with SNMP1 to elucidate whether they are indeed involved in pheromone detection. 

Comparing the three phylogenetically distinct groups of SgreORs revealed that in addition to most members of group I [24], a relatively large number of ORs from group II were expressed in SNMP1-positive cells (Figure 2, Figure 3 and Figure 4, Table 1). This aspect is remarkable because from a phylogenetic point of view, group I and group II are very different. Group I almost exclusively comprises SgreOR types with an orthologue in *L. migratoria*. The relatively high sequence identity observed between receptor types of this group from different locusts is reminiscent of pheromone receptors from several lepidopteran species that are supposed to mainly utilize orthologous ORs for pheromone detection [7,40,41,42]. By contrast, group II encompasses paralogous members as well as SgreORs with an orthologue in *L. migratoria* and non-paralogous SgreORs that lack such an orthologue (Figure 1). For ORs from group II, the present findings demonstrate that most SgreOR types from all these three categories of group II are co-expressed with SNMP1 (Figure 2, Figure 3 and Figure 4). This observation suggests that for members of group II, the affiliation to this particular group is more relevant in terms of co-expression with SNMP1 than the existence of paralogues or orthologues. Consequently, it is tempting to speculate that already ancient members from group II of locust ORs were co-expressed with SNMP1, and that this co-expression has been retained throughout evolution. With respect to pheromone detection, for SgreORs from both group I and group II that are co-expressed with SNMP1, one might also hypothesize that relevant ORs types with an orthologue in *L. migratoria* could be involved in perceiving pheromonal substances that are shared by different locust species. Although the chemical nature of locust pheromones is still a matter of controversial discussion [35,36], in line with this notion, volatiles released by locusts, such as guaiacol, phenol and the mixture of these with veratrole, have been proposed to function as “cohesion pheromones” in both *S. gregaria* and *L. migratoria* [43]. In contrast, paralogous ORs that are co-expressed with SNMP1 could be rather implicated in the detection of species-specific pheromone compounds. As an example, phenylacetonitrile (PAN, also called benzyl cyanide) is emitted by gregarious adult males of *S. gregaria* and appears to be involved in courtship inhibition and aggregation [35,36]. In *L. migratoria*, however, PAN does not serve as an aggregation pheromone [44], and so far, there is no evidence that it is involved in courtship inhibition in *L. migratoria*. Instead, it has been proposed that PAN contributes to antipredator defense mechanisms in *L. migratoria* [44]. 

Similar to group II, the branch of the phylogenetic tree harboring ORs from group III comprises receptors with paralogous or orthologues as well as non-paralogous SgreORs without an orthologue [24]. Yet, in marked contrast to group II, SgreORs from group III are barely co-expressed with SNMP1 (Figure 5 and Table 1). In view of the essential role of SNMP1 for pheromone detection in insects, this observation suggests that SgreORs from group III might not be of substantial relevance in terms of pheromone reception. 

In lepidopterans, many receptors for pheromones, notably sex pheromones, are expressed in a sex-specific or at least sex-biased manner [7,9]. However, in a recent study, regarding the antennal expression of candidate pheromone receptors belonging to group I of SgreORs, no obvious differences between male and female desert locusts were observed [33]. Likewise, in the present study, exploring possible differences in the expression of group II SgreORs between males and females, no evident discrepancies were detectable (Figure 6). Thus, the only minor differences in the expression levels of relevant ORs between male and female animals that were found in our recent [33] and present studies, might argue against the involvement of SNMP1-co-expressed receptors from group I and II in the detection of sex pheromones. However, it has to be considered that some sex pheromones that are released in a sex-specific manner are capable of regulating mating-related behaviors in males and females via a given pheromone receptor expressed in both sexes. For instance, in *D. melanogaster*, the male-specific pheromonal compound 11-cis-vaccenyl acetate affects both male and female mating behavior via the pheromone receptor Or67d. This OR type is essential in male and female vinegar flies to elicit the appropriate responses to 11-cis-vaccenyl acetate [45]. Analogously, it is conceivable that potential receptors for sex pheromone compounds in locusts may be expressed at similar levels in males and females. 

Based on morphological aspects, the olfactory sensilla of insects are classified into three major categories: coeloconic, trichoid and basiconic [46,47]. For pheromone detection, trichoid and basiconic sensilla are considered to be of particular relevance [reviewed in 25]. In *S. gregaria*, SNMP1 is only expressed in OSNs of trichoid or basiconic sensilla [28], which implies that the co-expression of SNMP1 and ORs is restricted to these two sensilla types. In line with this view, the vast majority of ORs from groups I and II are expressed in OSNs localized in the large neuronal cell clusters characteristic of basiconic sensilla, while three receptor types (OR3, OR102 and OR111) belonging to these two groups were found to be expressed in OSNs of trichoid sensilla [24,32]. 

## 5. Conclusions

In search for candidate pheromone receptors, we have analyzed about two-thirds of the *S. gregaria* OR repertoire for a possible antennal co-expression with the “sensory neuron membrane protein 1” (SNMP1), a marker for pheromone-responsive OSNs. In total, a relatively high number of 33 SgreORs were found to be co-expressed with SNMP1 in OSNs, suggesting a large repertoire of pheromone receptors in *S. gregaria*. Strikingly, almost all of the SNMP1-co-expressed OR types belong to the phylogenetically classified groups I and II of SgreORs. Moreover, most members of these two groups were observed to be co-expressed with SNMP1. By contrast, analyzing numerous SgreORs from group III, which comprises almost 60% of all SgreORs, only three receptor types were expressed in SNMP1-positive OSNs. Thus, receptors co-expressed with SNMP1 in *S. gregaria* are not evenly distributed throughout the entire OR repertoire, but cluster in distinct phylogenetic OR groups. In summary, our results indicate that in contrast to SgreORs from group III, a larger number of OR types from groups I and II could be involved in pheromone communication of *S. gregaria*.

## Figures and Tables

**Table 1:** Summary of SgreORs from group I-III that are co-expressed with SNMP1. The SgreORs are categorized in the relevant groups and indicated by the color code given in the schematic image of the phylogenetic tree depicted in Figure 1. In total, based on the results of FISH experiments, 33 out of the examined 83 SgreOR types are co-expressed in OSNs with SNMP1, whereas 50 SgreORs are not.

SgreOR Group	Co-Expressed with SNMP1				Not Co-Expressed with SNMP1			
**Group I (9 ORs)** **8 ORs examined**	**OR2** **OR8**	**OR3** **OR9**	**OR5**	**OR6**	**OR4**	**OR7**		
**Group II (38 ORs)** **33 ORs examined**	**OR84** **OR90** **OR94** **OR101** **OR109** **OR113**	**OR85** **OR91** **OR95** **OR102** **OR110** **OR114**	**OR86** **OR92** **OR96** **OR103** **OR111** **OR116**	**OR88** **OR93** **OR97** **OR108** **OR112** **OR118**	**OR82** **OR98** **OR106**	**OR83** **OR99**	**OR87** **OR100**	**OR89** **OR105**
**Group III (70 ORs)** **42 ORs examined**	**OR22**	**OR23**	**OR38**		**OR11** **OR16** **OR27** **OR31** **OR35** **OR41** **OR49** **OR54** **OR66** **OR71**	**OR13** **OR17** **OR28** **OR32** **OR37** **OR43** **OR51** **OR57** **OR67** **OR76**	**OR14** **OR25** **OR29** **OR33** **OR39** **OR45** **OR52** **OR61** **OR68** **OR80**	**OR15** **OR26** **OR30** **OR34** **OR40** **OR47** **OR53** **OR62** **OR70**

## Supplementary Materials

The following are available online at https://www.mdpi.com/2075-4450/10/10/350/s1, Figure S1: Expression of SNMP1 and group II SgreORs, Table S1: Oligonucleotides used for receptor cloning, Table S2: Oligonucleotides used to analyze the expression in male and female antennae of group II OR types co-expressed with SNMP1.
