# Supplementary material for: A Subset of Odorant Receptors from the Desert Locust Schistocerca gregaria Is Co-Expressed with the Sensory Neuron Membrane Protein 1
Source: Insects. 2019 Oct 17;10(10):350. doi: 10.3390/insects10100350 (PMC6835626; doi:10.3390/insects10100350)
Supplement: Supplementary file 1 [file insects-10-00350-s001.pdf]

Supp. Figures and Tables

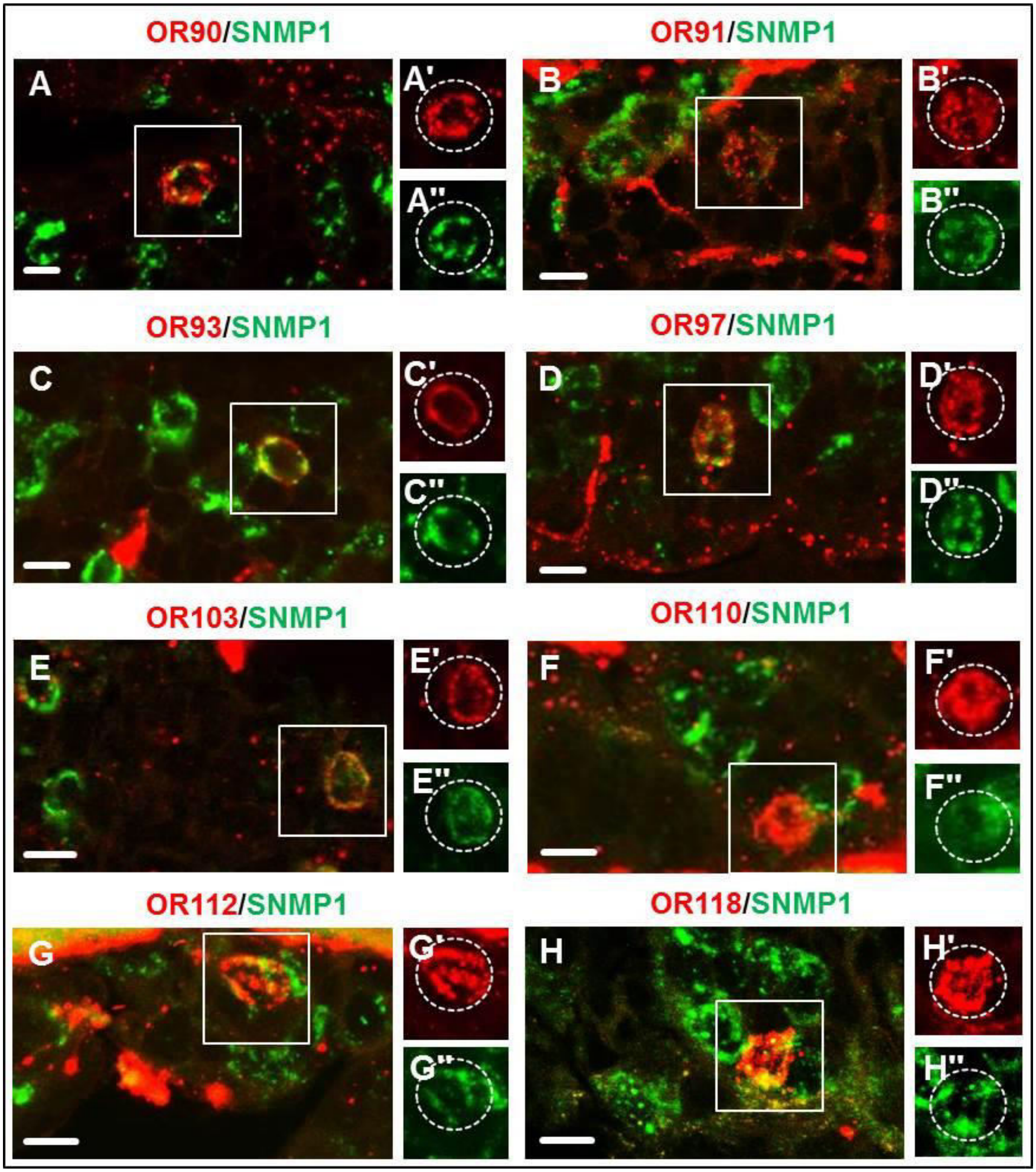

Fig. S1 Expression of SNMP1 and group II SgreORs.
(A-H) Expression of distinct group **II** OR types as well as SNMP1 was visualized by two-color FISH on antennal sections from *S. gregaria* using specific antisense riboprobes for SNMP1 (green) and SgreORs (red). (A′-H′, A′′-H′′) High magnification images of the

boxed areas in A-H are shown in either the red or the green fluorescence channel. The dashed lines highlight cells that express the defined SgreOR types. Scale bars, 10 μm.

Table S1

Oligonucleotides used for receptor cloning

OR ID	forward Primer（5' to 3' direction）	reverse Primer（5' to 3' direction）
SgreOR11	GACACAACGGCACCTGGCTCTTCC	ACGTCACAGCGTCGTAAGCTGCCAG
SgreOR13	CTCACACTGCTCAGCAGCAAAATTG	AGCAGCAGCGACCGTTTGATGGACA
SgreOR16	ACTGTTGGAAAACTGCACAGGAGGC	CACAATCGAGACGAAGGATTCCCTG
SgreOR22	GCTGCGGCATCGGATTTCTAGTAA	TCCTGATGAAGGCTTCCCTGGAGAG
SgreOR23	GTCTCGCTCCACCAGCGCATCATCA	GCAGCACAGTGAAGTATGAGTAGG
SgreOR25	ATGTGCTCTGGGCAGCCAGCTCACA	CAGTCGGAGTTGAGCTGCCTCAGCA
SgreOR26	GTGGTGATCCTGCTGCTGTTCGTGG	CGTGGAGTCAGCGTTCTTAGCGATG
SgreOR27	CATCTCGTGCATCGTGGACGCTTTC	GCTGATCTCCCTCAGTACCGCAAAG
SgreOR28	CTTCACGTCCCAGACGGCTTCTTC	CAGCAGCGCGAAGTACGAATAGGAG
SgreOR29	TCTTCACCACGCTCTGCTTCGCCTT	CGCTCTCCAAATAAACTTCGCCTCC
SgreOR30	GGTAATGGGACGTTGGAGAGACACC	GCTTCGTGCTGGGGGTCGTTCATCT
SgreOR32	CGTCCTGCTTCAGTTCACAGGAGCT	CCGTTCACCTGGTTGAGAAGAGCGT
SgreOR34	CCATCCTGAGCGCGTCGTACAAGTC	CTGTAGCGCCCAAGAACTGGAACAG
SgreOR37	CCTGTCGTGTGCTGGTACGGTTCGT	CCCATCCGCATACCAGCAGTACATG
SgreOR38	GATCACTGCCAACAATAGCATGGTATCC	CTGTGTCGTTTATTTGCCTCAGAACTGC
SgreOR40	CTTCTCGTTGATTTCCTCTGCTGC	CAGAGCAAAAGCGATGGCGAGGTT
SgreOR52	TCCGTCGAAGCCGTGATCCACATCA	TTGTCAGTCGCCGCAGCCTTACGAG
SgreOR54	AGTGCACCGCCACTTTCTTCTGCT	GAGTAGGTCACCTGCAAAAGCGAGA
SgreOR61	AACTGCTACGAGGGTTCTGAGGAAG	TAGCGGTTGTGGATGCGGCTCAGCA
SgreOR62	GTCCAGAGCGCGTCGTCCGTCATAT	CGAGGCATTCACCAGCGAGACAAAG
SgreOR66	GGGCGTGTGGAACGCAGTAGAGAGT	CATCGGGTAGAGGTGCGCAGCGGTG
SgreOR68	CATCCTACACGACTCCCGCAAACAT	GCACTAATGTCCTCACCGTATGTCG
SgreOR71	TCACGAAGGGGCTGCTACTGCTCAT	AGACCGCCTCTGCCTGTGCTGTTAC
SgreOR80	ATGGCATCCTTCGCCTTCTCGTCCA	CTCCCTGGAGAGGGGGAAGAACCCG
SgreOR88	ATGTCTCGCAGGATCAACCGCTGCT	AAACACCGTGCCACA AAGGTTAGCC
SgreOR89	ATGCCGGCCTTCGTCGCCCTCAAGC	TTTCCGAAAATGCAGTACAATCCCG
SgreOR90	AGACTGTGTGCTGATTTTCCAGAAG	TGCTTCGACAGCTGACACGTCTTT
SgreOR91	ATGGGAAAGCCTTCAAACGATCCTG	GTTGTGATTTGGCCGAAAAGACAG
SgreOR92	TTCTCAGCTCTCAAGCTTATCATG	CACCGTGATCTCCAGAGGCTTCTTG
SgreOR95	TTGGTCCCGGAGCGTCCATACGGCG	GAGGAGGCTCCTCTTGAAGCGGGCG
SgreOR96	GCCTGGCTGTCCATTTTCGCTGCCC	CACTCTGATCAGTTAAGATCTGCCC
SgreOR98	CTTTTTAGCAGCAATGGTGAGGTCG	TATTGCTGGATAATGCAATCGTCG

SgreOR101	CTTGCTGCTGTGCGCCTTCTACTCGG	TGTGCTATTCAGGCGATACAACATG
SgreOR102	CAGCGCCGCTACTGGGACTGCAGC	CTAGCTTTTCCGTTCCTTGTCGCTC
SgreOR103	CGCAGCTCAGTGCGTTGCCCTGAC	ATGGAAATAAATAGATTTCCGCAC
SgreOR106	CTGCCACAAGGACACTCCTCCAGCA	TGGTTCTTCTTCACCGACTGTTTGG
SgreOR107	ACAACCGGGAAGTGGGCCCATCCGC	AGAATCTGCAAGAAGGTCTCCCTGG
SgreOR108	GTGCACCAATTGTAGCTCCTGGACC	CCCTGATACTGAGGGGCTGTGACGC
SgreOR109	GATGACGCAAAGAGACGACTATACG	GAAAGAGTTTCTGGAGAGTGTGAAG
SgreOR110	ATGGTGTCGGTTCTAGTACGGCAGA	GATCATCATGTTGAAGATCGTGTAC
SgreOR111	ACACCATCA ACTGATTTTGGTCTAC	CTATTTCTGCACTCCGTGTAACATG
SgreOR112	CAGACCCTCATGTCGCCACCAGCCG	CTGCCGCTTGCAGATTAAACAGCAT
SgreOR113	GCACCAGAGGTAGATGCAGATAGTC	GAATGAGTTCCTGGAGAGCGTGACG
SgreOR114	GCGTCTTCGTGCTGCTGCTCAACCT	GAAGGTGCGCTTGGAGAGGGTGTAC
SgreOR116	TGGAGAGGGTGATGGGCCAGTCCAT	TCGAACAGCAGGTTGAACACCGTGT
SgreOR118	GATGTGCCGGTCGATAGTACAACGC	GATCCGTTCAGCACCCTGACGAAGG

Table S2

Oligonucleotides used to analyze the expression in male and female antennae of group II OR types co-expressed with SNMP1.

OR ID	forward primer（5’ to 3’ direction）	reverse primer（5’ to 3’ direction）
SgreOR84	GTCCCGAATCGTCACGAAGACCGAC	TCAGTGCATACTGTACAGCATATTC
SgreOR85	CTGTGCAGCCTTGTCGCTACTATCG	CTGGAGCACAGGTTTGCCATGTTG
SgreOR86	GGCTACGGTTCTGCAGTGAAGATAG	GTTTGAAGCGAGCGTCACTGTTGAC
SgreOR88	GTTGTCTCAGGCCACTGTATGCATG	TCTCGAGCGGACGCATCGCTGCCAC
SgreOR90	AGACTGTGTGCTGATTTTCCAGAAG	TGCTTCGACAGCTGACACGTCTTT
SgreOR91	ATGGGAAAGCCTTCAAACGATCCTG	GTTGTGATTTGGCCGAAAAGACAG
SgreOR92	TTCTCAGCTCTCAAGCTTATCATG	CACCGTGATCTCCAGAGGCTTCTTG
SgreOR93	CTCTACGGTGTTCAATCTTTCAGCG	CATAAGATCCATTCAGCACCTGCAC
SgreOR94	TTATGGCAACGGCAAAAGCAGTCAC	GCGTCGCAGTCTGCCCAGTCACAGC
SgreOR95	TGCTCGCCAGTTCCCCGTACCACTG	GCCCACCGTGATCTCCAGAGGCCG
SgreOR96	ATCCGACTCCGAAACCAGAAGCTGG	CAGCTTCTCACTCTGATCAGTTAAG
SgreOR97	CAGGTGTCGACAATGGTGGCCTCT	GAGAGTTTGCAGGTTTTCCCCACAG
SgreOR101	CTTGCTGTCGTGCGCCTTCTACTCGG	TGTGCTATTCAGGCGATACAACATG
SgreOR102	GTGGTCGGTGCAGCCGCTGATCTC	CATTCAGCACCTGCACGAACGTGG
SgreOR103	CGCAGCTCAGTGCGTTGCCCTGAC	ATGGAAATAAATAGATTTCCGCAC
SgreOR109	AAGATGAACATTCAACACCATCAGG	GAATATTGTGTACGAAACGTTCATG
SgreOR110	GATCGTACTCCTTGTTAGCGTTGTC	CATCATGTTGAAGATCGTGTACGATG
SgreOR111	AATCGATCAGAGTGAGCGCCTGGTG	CTATTTCTGCACTCCGTGTAACATG
SgreOR112	TACTGGAAGCTACACAGCAGTTTAG	ATGAATTCCTGGACAGCTCGACGAG
SgreOR113	TCAGCGGATATTTAGTTGCACCAG	TTAGTGCCTGCAGGAATGAGTTCC
SgreOR114	GCTGCAGGTGTTCACAATCGTCGTC	GAGGAAGGTGCGCTTGGAGAGGGTG
SgreOR116	GACCAAGATGCTCTTCACGTTCGTC	CAGGTTGAACACCGTGTAGGACATG
SgreOR118	GTCACTGCTAGCCTGCTGCAAAGTG	GATCCGTTCAGCACCCTGACGAAGG

actin	ATGGCTACTGCTGCATCCTC	CACATCTGCTGGAAGGTGGA
